# Psychedelic Therapies for Comorbid Major Depressive Disorder and Chronic Pain: A Review of Putative Mechanisms of Action

**DOI:** 10.1002/prp2.70238

**Published:** 2026-04-06

**Authors:** Jordana Kazdan, Karim S. Ladha, M. Ishrat Husain

**Affiliations:** ^1^ Temerty Centre for Therapeutic Brain Intervention Centre for Addiction and Mental Health Toronto Ontario Canada; ^2^ Pharmacology and Toxicology, Temerty Faculty of Medicine University of Toronto Toronto Ontario Canada; ^3^ Department of Anesthesiology and Pain Medicine University of Toronto Toronto Ontario Canada; ^4^ Department of Anesthesia Women's College Hospital Toronto Ontario Canada; ^5^ Krembil Brain Institute and Centre for Mental Health United Health Network Toronto Ontario Canada; ^6^ Department of Psychiatry, Temerty Faculty of Medicine University of Toronto Toronto Ontario Canada

**Keywords:** chronic pain, depression, LSD, MDD, mood, psilocybin, psychedelics

## Abstract

Major Depressive Disorder (MDD) and chronic pain are independently debilitating conditions that frequently co‐occur. This comorbidity poses a significant clinical challenge, resulting in greater symptom severity, higher disability, and worse prognosis than either condition alone. Current therapies often address each disorder in isolation, leaving individuals with comorbid MDD and chronic pain underserved. Serotonergic psychedelics such as psilocybin, N,N‐dimethyltryptamine (DMT), and Lysergic Acid Diethylamide (LSD) have reemerged as promising therapeutic targets for a range of neuropsychiatric disorders. When combined with psychological support, psychedelics show rapid and sustained antidepressant potential, and preliminary evidence supports analgesic effects. Despite substantial overlap in the biological and psychological processes underlying MDD and chronic pain, research on psychedelics for this comorbidity remains largely unexplored. This narrative review examines putative mechanisms through which psychedelics target symptoms of both MDD and chronic pain. Mechanisms considered include serotonergic modulation via the 5‐HT2A receptor, anti‐inflammatory effects, neuroplastic changes, altered brain network dynamics, psychological effects, and the influence of set and setting. While most existing evidence comes from populations with either depression or pain alone, the breadth of proposed mechanisms supports psychedelics as a unified therapeutic approach for comorbid MDD and chronic pain. This review provides a compelling rationale for future clinical trials to evaluate psychedelic‐assisted therapies for complex neuropsychiatric and medical conditions.

**Abbreviations**
5‐HT: 5‐hydroxytryptamine
ACC: anterior cingulate cortex
ACT: acceptance and commitment therapy
BDNF: brain‐derived neurotrophic factor
CBT: cognitive–behavioral therapy
DMN: default mode network
dmPFC: dorsomedial Prefrontal Cortex
DMT: n,n‐dimethyltryptamine
ECT: electroconvulsive therapy
EN: executive network
IL: interleukin
LSD: lysergic acid diethylamide
MAO‐A: monoamine oxidase‐A
MBCT: mindfulness‐based cognitive therapy
MBSR: mindfulness‐based stress reduction
MDD: major depressive disorder
mPFC: medial prefrontal cortex
mTOR: mammalian target of rapamycin
PCC: posterior cingulate cortex
PET: positron emission tomography
PFC: prefrontal cortex
RCTs: randomized clinical trials
SMA: supplementary motor area
SN: salience network
TNF‐α: tumor necrosis factor‐alpha
TRD: treatment‐resistant depression
TrkB: tropomyosin receptor kinase B
vlPFC: ventrolateral prefrontal cortex

## 1 Introduction

Major depressive disorder (MDD) and chronic pain are highly prevalent, debilitating conditions affecting millions of people worldwide [1]. Depressive and pain disorders share a well‐established bidirectional relationship where experiencing symptoms of one condition exacerbates the risk of developing the other [2]. Approximately two‐thirds of patients diagnosed with MDD present with unexplained pain [2, 3]. Similarly, over half of individuals with pain disorders meet the diagnostic criteria for MDD [2]. Compared to either condition alone, comorbid MDD and chronic pain are associated with worse prognosis, greater symptom severity, prolonged duration of illness, higher healthcare utilization, and increased suicide risk [2, 4, 5]. The heterogenous nature and varied clinical expression of both MDD and chronic pain disorders further complicate diagnosis and symptom management. This comorbidity predicts poorer response to standard treatments, contributing to treatment resistance and chronic illness [5, 6].

In recent years, there has been a resurgence of interest in the therapeutic potential of serotonergic psychedelics, such as psilocybin and lysergic acid diethylamide (LSD), for the treatment of neuropsychiatric disorders. When combined with psychological support, psychedelics elicit profound changes in consciousness that are associated with improved mood and well‐being across several psychiatric conditions [7]. Many participants in randomized clinical trials (RCTs) of psychedelic therapies describe their experience as extremely meaningful, with themes of emotional breakthroughs, a deepened connection to self, an openness to new perspectives, and a renewed confidence to overcome life obstacles [8, 9, 10]. A recent meta‐analysis reported that psilocybin therapy has robust antidepressant effects, with greater reductions in depressive symptoms and remission rates compared to controls [11]. LSD and ayahuasca have also shown sustained antidepressant effects in RCTs [12, 13].

While psychedelic research has primarily focused on treatment for mental and substance use disorders, interest in their potential for treating pain is gaining momentum. A recent review synthesized results of 26 studies investigating the use of psychedelics in pain management, highlighting their therapeutic potential for fibromyalgia, chronic neuropathic pain, cluster headache, and migraine, phantom limb pain, and cancer‐related pain [14]. However, despite substantial evidence demonstrating shared neurobiological and psychological therapeutic targets in depression and pain, only two ongoing trials are investigating the clinical potential of psychedelics in the comorbid conditions (NCT06518720 and NCT06355414).

Given the limited clinical investigation in this area, there is a need to better understand how psychedelics may act on shared pathways implicated in both MDD and pain. In this context, this narrative review explores the putative mechanisms by which psychedelic therapies may alleviate symptoms of comorbid MDD and chronic pain, drawing from current theories and emerging evidence from preclinical and clinical studies.

## 2 Methods

A literature review was conducted using electronic searches of Medline and Google Scholar for articles related to serotonergic psychedelics, depression, and pain. Serotonergic psychedelics included were psilocybin, LSD, n,n‐dimethyltryptamine (DMT), and other substances primarily acting on the serotonergic 5‐hydroxytryptamine (5‐HT) 2A receptor. Search terms included “psychedelics,” “psilocybin,” “LSD,” “Ayahuasca,” “mechanisms of action,” “depression,” and “chronic pain.” Searches were unrestricted by publication date but limited to English‐language articles. References from included articles and recent reviews were also screened for additional relevant studies.

Given the breadth of the topic and the evolving nature of the field, studies were selected based on their relevance to the conceptual framework of the review rather than through predefined inclusion or exclusion criteria. Both preclinical and clinical studies were considered, with a focus on mechanistic findings. Priority was given to studies examining comorbid depression and chronic pain, or findings translatable across both conditions.

## 3 Pharmacology of Psychedelic Drugs

Serotonergic psychedelics are categorized into three classes based on their chemical structure: tryptamines (psilocybin, ayahuasca, n,n‐dimethyltryptamine (DMT)), phenethylamines (mescaline), and ergolines (LSD) (Table 1).

**TABLE 1:** Comparative pharmacology of psychedelics.

Compound	Chemical class	Primary receptor targets	Onset/Duration
Psilocin (from Psilocybin oral capsule)	Tryptamine	5‐HT2A (partial agonist), 5‐HT1A, 5‐HT2C [15]	2.5 h half‐life; ~2 h peak onset; 4–6 h duration [16]
DMT (IV)	Tryptamine	5‐HT2A (full agonist), 5‐HT1A, 5‐HT2C [15]	8 min half‐life; 2–5 min peak onset; 20–30 min duration [17]
DMT (ayahuasca herbal tea)	Tryptamine	5‐HT2A (full agonist), 5‐HT1A, 5‐HT2C [15]	3–5 h half‐life; 1.5–2 peak onset; 4–6 h duration [17]
Mescaline (oral capsule)	Phenethylamine	5‐HT2A (weak, partial agonist), 5‐HT1A, 5‐HT2C [15]	3.5 h half‐life; 2 h peak onset; 10–12 h duration [16]
LSD (oral solution)	Ergoline	5‐HT2A (partial agonist, high affinity), 5‐HT1A (high affinity), dopaminergic, adrenergic [15]	3.5 h half‐life; 90 min peak onset; 8–12 h duration [16]

### 3.1 Tryptamines

Tryptamines share chemical similarities with the endogenous neurotransmitter serotonin. Psilocybin is a psychoactive compound found in hundreds of mushroom species. It is a prodrug for the pharmacologically active metabolite psilocin, which is a non‐selective, partial agonist at 5‐HT2A/2C/1A receptors [15]. Ayahuasca is traditionally prepared as an herbal tea with plants containing DMT and β‐carboline alkaloids, including harmine. β‐carbolines inhibit monoamine oxidase‐A (MAO‐A), preventing DMT degradation and enabling activation of 5‐HT2A/2C/1A receptors [12]. DMT binds to 5‐HT2A with lower affinity than psilocin; however, it acts as a full agonist with higher activation potency, aligning with its intense and rapid psychedelic effects [15]. Harmine may also produce antidepressant effects outside of MAO‐A inhibition [12].

### 3.2 Phenethylamines

Mescaline is a natural compound found in peyote and San Pedro cacti. It shares a similar chemical structure to norepinephrine, epinephrine, and dopamine [18]. Mescaline exhibits the least potent interaction with serotonin receptors among serotonergic psychedelics [15]. Compared to other psychedelics, its therapeutic potential remains largely unexplored.

### 3.3 Ergolines

LSD is a semi‐synthetic compound derived from ergot alkaloids with the highest binding affinity for 5‐HT2A and 5‐HT1A receptors, as well as other 5‐HT1 receptor subtypes [15]. Unlike tryptamines, LSD also interacts with dopaminergic and adrenergic receptors, which may contribute to its unique psychoactive and euphoric properties [15].

## 4 Mechanisms of Therapeutic Action

**FIGURE 1:**
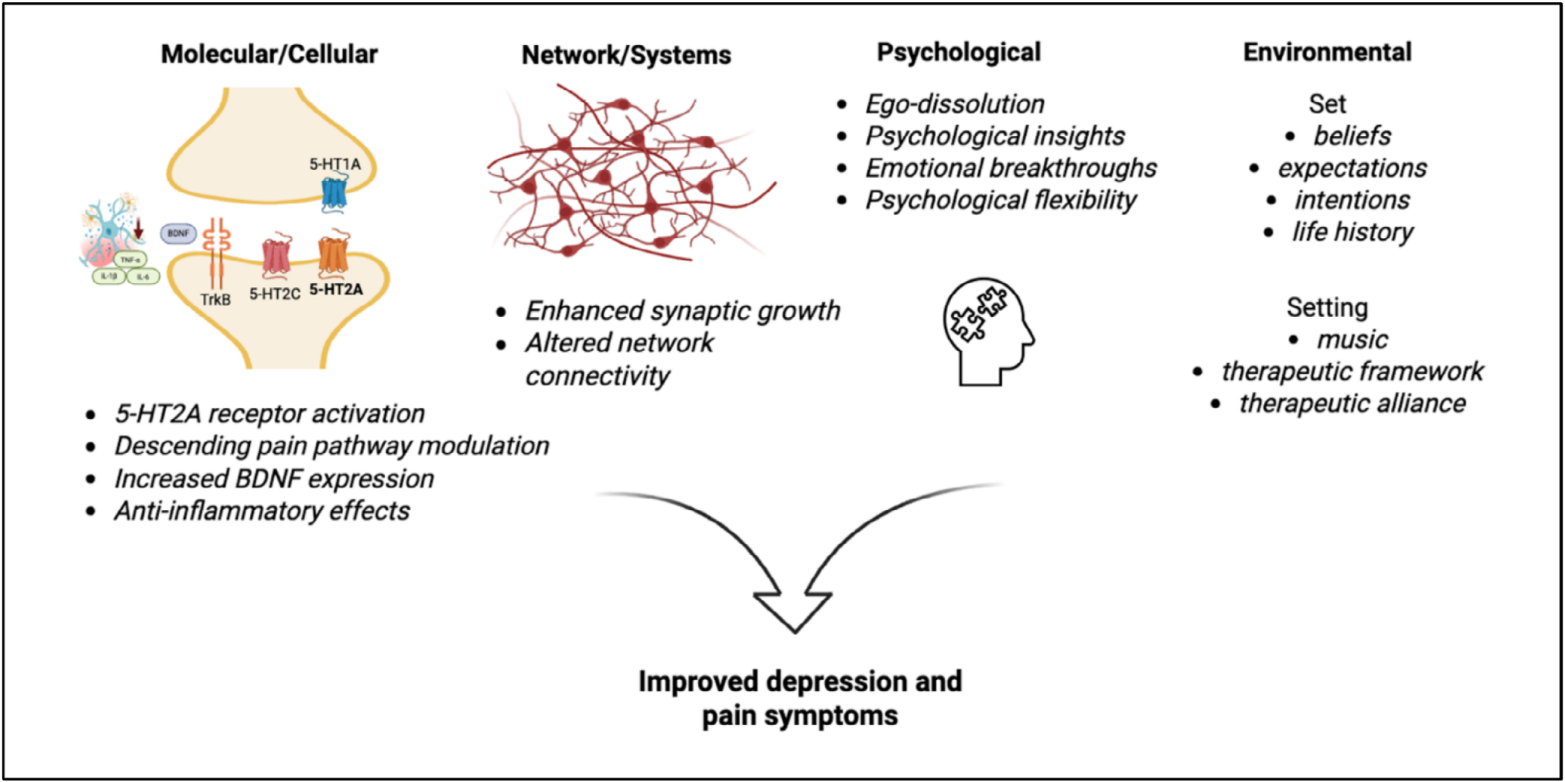
Psychedelic mechanisms in depression and pain.

Several mechanisms have been proposed to contribute to the therapeutic effects of serotonergic psychedelics. The current evidence suggests that psychedelics may improve symptoms of depression and pain through a dynamic interplay between molecular targets, neural network connectivity, psychological processes, and environmental factors (Figure 1).

### 4.1 Molecular

#### 4.1.1 5‐HT2A Receptors

The acute, psychoactive effects of serotonergic psychedelics are largely mediated through their action at the 5‐HT2A receptor [19]. 5‐HT2A receptors are widely distributed throughout the brain and densely expressed in regions associated with mood and pain processing, including the prefrontal cortex (PFC), anterior cingulate cortex (ACC), and posterior cingulate cortex (PCC) [19, 20]. These receptors are primarily located on layer V pyramidal neurons, which serve as the principal output neurons of the cortex. Activation of 5‐HT2A receptors triggers a surge in glutamate and cortical excitability that alters neural network activity [19]. Several neuroimaging studies have demonstrated that psilocybin and LSD increase thalamic connectivity with primary sensory regions while decreasing connectivity with associative networks, thereby altering how information is processed and integrated [21, 22]. This shift in connectivity is believed to facilitate psychoactive effects and adaptive cognitive change. 5‐HT2A receptor involvement is strongly supported by evidence that psychedelic‐induced cortical excitability, neural network changes, and psychoactive effects are all reliably blocked by 5‐HT2A antagonists [19, 22].

Beyond acute psychoactive effects, 5‐HT2A agonism engages several downstream neurophysiological processes implicated in antidepressant and nociceptive outcomes. Among these processes are inflammation and brain‐derived neurotrophic factor (BDNF) signaling pathways, which are discussed in later sections.

In pain modulation, the role of 5‐HT receptors is highly complex and context‐dependent [23]. 5‐HT receptors modulate pain through the descending inhibitory circuit, which projects from brainstem regions to the spinal cord [23]. Evidence from preclinical studies suggests that activation of the spinal 5‐HT2A receptors contributes to pain relief caused by nerve injury [24]. On the other hand, upregulation of 5‐HT2A receptors in the spinal cord can lead to hypersensitivity and has been observed in certain inflammatory and neuropathic pain conditions [18, 24]. In rodent models of pain, ayahuasca was shown to interact with the descending pain pathway, producing dose‐dependent antinociceptive effects [25]. A potential reason for these contradictory effects may be explained by evidence indicating that psychedelics rapidly induce downregulation and desensitization of 5‐HT2A receptors [26].

The downregulation hypothesis is consistent with clinically effective antidepressant strategies such as augmenting selective serotonin reuptake inhibitors (SSRIs) and serotonin‐norepinephrine reuptake inhibitors (SNRIs) with second‐generation antipsychotics [27]. These agents have also been shown to exhibit analgesic properties and are prescribed as first‐line treatments for a range of chronic pain conditions [27, 28]. A shared pharmacological action among these agents is their functional antagonism of 5‐HT2A receptors and paradoxical 5‐HT2A downregulation after chronic administration [27, 29, 30]. While data supporting this model are largely theoretical, regulating serotonin signaling via the 5‐HT2A receptor may serve as a shared mechanism for treating both MDD and pain.

#### 4.1.2 Other Receptors

While the 5‐HT2A receptor has received the most attention, other receptor systems may also contribute to the therapeutic mechanisms of psychedelics. In particular, 5‐HT1A receptors are highly expressed in brain regions involved in mood and pain regulation [31]. After chronic administration, LSD has been shown to desensitize 5‐HT1A receptors in a manner similar to SSRIs, which has been correlated with anti‐stress properties [32]. LSD has also been shown to interact with the 5‐HT1A receptors involved in the descending pain inhibitory pathway [33]. Given its involvement in both affective and nociceptive processing, the 5‐HT1A receptor represents a promising target in psychedelic research and merits further investigation.

In addition, LSD has been shown to interact directly with dopamine D1 and D2 receptors in the mesolimbic system, a network associated with reward processing, emotional response, and motivated behavior [32, 34]. Dysregulation of mesolimbic dopamine has been implicated in both MDD and chronic pain, yet its role in psychedelic therapy remains unexplored [35, 36]. Future research should investigate whether LSD's dopaminergic effects contribute to motivational and functional improvements in comorbid depression and chronic pain.

### 4.2 Inflammation

Inflammation is increasingly recognized in the etiology of MDD and chronic pain, possibly contributing to their reciprocal relationship [37]. Elevated levels of pro‐inflammatory cytokines such as interleukin (IL)‐1β, IL‐6, and tumor necrosis factor‐alpha (TNF‐α) are consistently observed in individuals with MDD [38, 39]. In healthy volunteers, experimentally induced inflammation increases depressive symptoms [40]. Inflammation in the peripheral and central nervous system is implicated in several chronic pain conditions including arthritis, fibromyalgia, and complex regional pain symptom [41, 42, 43]. Several studies have correlated elevated levels of cytokines, including IL‐6, IL‐8, TNF‐α, and C‐reactive protein, with increased pain sensitivity and nociceptor sensitization [44]. Inflammation may contribute to MDD and pain by disrupting synaptic plasticity [45, 46]. Pathological levels of pro‐inflammatory cytokines have been shown to reduce the expression of BDNF, a protein that regulates neuronal growth, survival, and synaptic remodeling [37, 45, 47].

Growing evidence suggests that psychedelics disrupt pro‐inflammatory pathways via 5‐HT2A receptor agonism [48]. Psychedelics have been shown to suppress TNF‐α‐induced increases in IL‐6, effects that were reversed by 5‐HT2A antagonists [49]. Since 5‐HT2A activation is typically pro‐inflammatory, psychedelics' anti‐inflammatory properties may be related to functional selectivity mechanisms [48, 50]. There is also emerging evidence that psychedelics' anti‐inflammatory effects involve sigma‐1 receptors, chaperone proteins expressed in neurons and immune cells [51, 52]. In vitro, DMT‐mediated sigma‐1 activation decreased levels of pro‐inflammatory cytokines (IL‐1β, IL‐6, TNF‐α, IL‐8) and upregulated the production of the anti‐inflammatory cytokine IL‐10 [52]. In preclinical models, psychedelics' immunomodulatory effects appear to be context dependent. A recent systematic review showed that psychedelics tend to exert pro‐inflammatory effects under normal physiological states and anti‐inflammatory effects under pathological states [48]. Clinical data on the inflammatory effects of psychedelics remain limited and mixed [48]. While interest in this field is growing, further research is required to confirm these effects as mechanisms of action of psychedelic therapies.

### 4.3 BDNF

MDD and chronic pain have been shown to exhibit structural and functional abnormalities in brain regions associated with mood and pain regulation [53]. BDNF regulates neurogenesis and synaptic plasticity through several signaling cascades, including tropomyosin receptor kinase B (TrkB) and mammalian target of rapamycin (mTOR) pathways [14, 54]. Several studies have reported lower BDNF levels in patients with MDD, and antidepressant treatment has been shown to increase BDNF levels [55]. Conversely, various pain conditions are associated with elevated BDNF levels [56]. Upregulation of BDNF–TrkB signaling has been shown to impair pain‐modulating processes and promote maladaptive neuroplasticity that sustains chronic pain sensitization [57, 58].

Psychedelics may facilitate improvements in mood and pain through restoring adaptive neuroplastic change [47]. Activation of 5‐HT2A receptors stimulates the release of BDNF [47]. In healthy controls and patients with treatment‐resistant depression (TRD), a single dose of ayahuasca led to increased BNDF levels compared to placebo, which correlated to improved depressive symptoms [59]. Psychedelics may also modulate BDNF expression and synaptic plasticity through non‐serotonergic pathways. DMT can bind to sigma‐1 receptors, and in preclinical studies, activation of this receptor has been shown to increase BDNF expression and produce rapid antidepressant‐like effects [52, 60]. Further, many antidepressants bind allosterically to TrkB, which has been shown to facilitate BDNF action and mediate their neuroplastic and behavioral responses [61]. Preclinical research has demonstrated that LSD and psilocin can bind to TrkB with much higher affinity than traditional antidepressants, possibly explaining their rapid antidepressant onset [62]. In addition, other neuromodulating interventions such as electroconvulsive therapy (ECT) have demonstrated both antidepressant and analgesic effects [63]. In patients with MDD, ECT increased BDNF levels in responders, but not non‐responders [64]. Although these findings suggest that the therapeutic benefits of psychedelics may be driven by BDNF‐related neuromodulator changes, this interpretation remains speculative. A meta‐analysis reported no significant changes in peripheral BDNF levels after a single dose psychedelic administration [65]. The authors suggest that peripheral BDNF levels in humans may not be a suitable biomarker for the rapid, neuroplastic effects seen in preclinical studies. No published chronic pain trial to date has directly linked psychedelic‐induced BDNF changes (central or peripheral) to clinical pain outcomes. Further research is needed to elucidate the effects of psychedelic‐induced BDNF levels on pain and depression symptomology.

### 4.4 Brain Connectivity

The Default Mode Network (DMN) is a network of brain regions that includes the medial prefrontal cortex (mPFC), posterior cingulate cortex (PCC), precuneus, angular gyrus, and hippocampus. The DMN is associated with high‐order processing, including self‐representation, memory, and the affective dimension of pain [66, 67]. Both MDD and chronic pain populations have been shown to exhibit increased hyper‐connectivity within the DMN that correlates with rumination and negative self‐focus [68, 69]. In patients with dysthymic disorder, antidepressant treatment with duloxetine normalized hyperactive DMN connectivity but not with placebo [70]. In patients with fibromyalgia, activation of the PCC has been associated with catastrophizing and translating the cognitive‐emotional processing of pain into heightened pain perception [71]. Further, several studies have observed that various chronic pain conditions exhibit abnormal connectivity between the DMN and insula, an area involved in integrating the sensory, affective, and cognitive aspects of pain perception [72, 73, 74]. In a study examining several pain populations, mPFC and insula connectivity was associated with increased pain intensity, and DMN‐insula coupling was related to decreased connectivity between the pPFC and other parts of the DMN [73]. Since the insula is implicated in the transition from acute to chronic pain, these findings suggest that pain becomes maladaptively encoded into the DMN [73, 74]. In a placebo‐controlled study in healthy volunteers, psilocybin reduced functional connectivity between the DMN and the right insula [75]. While interesting, these interpretations remain speculative. More research is needed to elucidate the relationship between psychedelics and neuroplastic effects within the DMN and insula in pain management.

Several neuroimaging and electrophysiological studies suggest that psilocybin, ayahuasca, and LSD transiently decrease functional connectivity within the DMN while increasing global connectivity [76]. By disrupting normal brain network activity, psychedelics alter how information is perceived, processed, and stored [22]. In this altered state of consciousness, individuals may have the capacity to engage with their thoughts in ways that promote constructive psychological and behavioral change, particularly when combined with psychological support. This assumption aligns with previous neuroimaging studies that suggest the DMN adaptively reorganizes following psychedelic therapy. In an open‐label trial, psilocybin therapy was associated with rapid antidepressant effects that were accompanied by decreased within‐network connectivity in the DMN and increased integration between the DMN and higher order brain networks such as the executive network (EN) and salience network (SN) [77]. A double‐blind RCT observed that following psilocybin therapy, changes in dynamic network flexibility, particularly within the EN, were strongly correlated with improvements in depression scores 6 weeks posttreatment [77]. These associations were not found for escitalopram, suggesting psychedelic‐specific mechanisms [77]. Taken together, these findings support a time‐dependent model in which psilocybin therapy initially destabilizes maladaptive network patterns, followed by longer‐term reintegration and increased neural adaptability, which may contribute to sustained therapeutic outcomes.

### 4.5 Altered State of Consciousness and Psychological Flexibility

Psychedelics are well known for producing profound changes in consciousness experienced through visual effects, distortions of time and space, a sense of awe, heightened emotions, and a unitive experience [78]. Among these subjective effects is the experience of ego‐dissolution, a ubiquitous psychedelic phenomenon in which the boundaries of the self are blurred and cognitive processes are decoupled from self‐referential thinking [76]. Several self‐referential processes, including a sense of agency, catastrophizing, fear‐avoidant beliefs, and self‐efficacy, have been shown to exacerbate symptoms and mediate the relationship between depression and pain [28]. By temporarily disrupting self‐related top‐down processing, psychedelics may create a window for individuals to reevaluate their beliefs with greater clarity and emotional detachment, allowing for emotional breakthroughs and psychological insights [79, 80].

The capacity to reassess rigid, self‐referential patterns appears to facilitate a shift toward psychological flexibility, an important mediator of both depression and pain severity [81, 82]. Psychological flexibility has been shown to mediate the relationship between the acute psychoactive effects of psychedelics and positive therapeutic outcomes [82, 83]. In patients with TRD who underwent psilocybin therapy, improvements in psychological flexibility were significantly correlated with reductions in depression severity [84].

The role of altered consciousness in facilitating therapeutic outcomes has been noted in pain research since the earliest studies of psychedelics. Scientists from the 1960s observed that a single, psychoactive dose of LSD (100 μg) produced significant and longer‐lasting pain relief compared to opioids, with nearly 50% of patients remaining pain‐free beyond 19 h [85]. The authors theorized that the pain relief was caused by a shift in attention away from the “ailing part” of the body [86]. This theory aligns with current research reporting that hallucinogenic doses are more effective in relieving pain than non‐hallucinogenic doses [87, 88]. However, despite promising evidence, the role of psychoactive effects in both antidepressant and pain treatment remains a subject of ongoing investigation [89]. While some studies report that mystical effects are a predictor of improved outcomes, there is emerging evidence suggesting antidepressant potential and analgesic effects with low and non‐hallucinogenic doses, warranting further investigation [90, 91, 92, 93].

### 4.6 Set and Setting

The therapeutic benefits of psychedelic therapy involve mechanisms beyond its pharmacological action. A central feature of psychedelic therapy is “set and setting”, a concept that emphasizes that an individual's mindset and environment can greatly shape the subjective effects of psychedelics [94].

*Set* refers to the internal state of the participant, including their life history, expectations, intentions, and current psychological state. *Setting* encompasses the external environment, both physical (e.g., art, music, seating, and presence of others) and psychosocial (e.g., the therapeutic relationship and interpersonal dynamics). Set and setting may provide context for the considerable variability in individual responses to psychedelics. For instance, LSD and psilocybin have been associated with profoundly positive experiences of joy, social connectedness, and psychological insight, but can also trigger feelings of anxiety, fear, or distress [95].

#### 4.6.1 Environmental

Most psychedelic studies acknowledge the influence of context‐driven mechanisms on therapeutic outcomes. Since emphasis on context‐driven mechanisms originates from indigenous psychedelic contexts, observational and real‐world evidence from naturalistic ceremonies provide important insights into potential therapeutic mechanisms. A study examining six placebo‐controlled, naturalistic ayahuasca ceremonies reported no between‐group differences in ratings of altered consciousness, and both groups experienced reductions in stress, anxiety, and depression post‐intervention [96]. The authors speculated that these results were, in part, driven by environmental and psychosocial factors associated with the ceremonies [96]. Another study reported that more positive ratings of the ayahuasca ceremonial setting correlated with more positive ratings of mystical experiences and lower incidences of challenging experiences [97]. In clinical settings, environmental factors are carefully considered to optimize psychedelic dosing rooms. Guidelines for psychedelic trials recommend designing an aesthetically pleasing space resembling a comfortable living room, including soft lighting, comfortable seating, pleasant imagery, plants, and music [98]. While there is consensus on the importance of set and setting in modern clinical research, there is limited direct evidence on how these factors affect therapeutic outcomes.

#### 4.6.2 Music

Music has well‐established benefits for both depression and chronic pain [99, 100, 101]. In psychedelic therapy, music sits at the intersection of *set* and *setting*. Participants in psychedelic studies often report that music evokes specific emotions, thoughts, and memories, guiding them through different psychological states [8, 102]. Psychedelics, in turn, can heighten the emotional and perceptual response to music. Psilocybin therapy has been shown to enhance music‐evoked pleasure, which correlates with later improvements in anhedonia [103]. During psilocybin therapy for depression, the extent to which participants resonated with the music and were open to the experience predicted mystical experiences and insightfulness [102]. Notably, these music‐related variables, but not drug intensity, predicted reductions in depression 1 week posttreatment [102].

The interaction between psychedelics and music is evidenced at a neurobiological level. Several studies have shown that psilocybin and LSD modulate neural responses to music through processes involving 5‐HT2A receptor signaling [104, 105, 106]. Increased brain responsiveness to music has been shown to correspond to the intensity of subjective effects [105, 106]. These music‐evoked neural changes may facilitate adaptive meaning‐making processes during psychedelic therapy. Under LSD, music enhances connectivity between the parahippocampal cortex and the visual cortex, promoting vivid, autobiographical mental imagery [107]. In a double‐blind randomized trial, LSD was shown to increase the perceived meaningfulness of music, which is associated with increased activity in brain regions involved in self‐referential and emotional processing, including the supplementary motor area (SMA), dmPFC, and vlPFC [108]. Collectively, current evidence suggests that music in combination with psychedelics can elicit powerful emotional experiences that contribute to therapeutic outcomes. Future research should aim to isolate music's therapeutic influence and clarify the mechanisms by which music enhances psychedelic experiences.

#### 4.6.3 Psychological Support

Most clinical trials of psychedelics involve a degree of psychological support that is usually based on a structured therapeutic model drawing from evidence‐based therapies such as cognitive–behavioral therapy (CBT), mindfulness‐based therapies (e.g., MBSR/MBCT), acceptance and commitment therapy (ACT), and emotionally supportive care [109, 110, 111, 112, 113]. Recent reviews show that CBT, MBSR/MBCT, and ACT reliably reduce symptom severity and improve function in both depression and chronic pain [114, 115, 116, 117]. Further, CBT, MBSR/MBCT, and ACT have been shown to improve measures of psychological flexibility, rumination, and catastrophizing [118, 119, 120, 121]. These shared mechanisms between evidence‐based interventions and psychedelic therapy reflect a potential synergy that may enhance therapeutic outcomes.

In addition to the structure of the psychological support, the quality of the relationship between the therapist and participant can influence the psychedelic experience. The therapeutic relationship is a well‐established predictor of clinical outcomes across a variety of psychotherapies [122, 123]. Psychedelics may facilitate the development of a strong therapeutic bond by regulating emotional responses and enhancing psychosocial traits such as empathy, openness, and social connectedness [124, 125, 126, 127, 128]. Multiple studies of psilocybin therapy for MDD reported that a stronger therapeutic relationship predicted both mystical experiences and emotional breakthroughs, which were associated with sustained improvements in clinical outcomes [129, 130].

A central role of psychedelic therapists is to manage participants' expectations and intentions before psychedelic dosing sessions. Participants' expectations have been shown to predict the intensity of acute psychedelic effects, which are associated with long‐term changes in mood and well‐being [131]. Further, participants' intentions prior to psychedelic experiences have been shown to affect therapeutic outcomes. For example, in a survey‐based study, sub‐hallucinogenic doses of psychedelics were reported to be more effective in relieving pain when pain management was a primary goal [88].

Expectations and intentions elicit measurable neurobiological and clinical responses across various diseases [95]. Importantly, placebo studies on patients with depression and pain have reported overlapping neurobiological mechanisms associated with treatment outcomes. Positron Emission Tomography (PET) imaging studies have demonstrated that placebo‐induced activation of the endogenous opioid system is associated with both antidepressant and analgesic effects [132]. This mechanism has also been linked to dopaminergic and reward neurocircuitry [133]. While findings drawn from placebo research are interesting, few studies directly assess these mechanisms in psychedelic clinical trials. Future investigation into set and setting should be conducted to leverage potential neurobiological placebo responses.

This schematic depicts several mechanisms of action of psychedelics that may interact to improve depression and pain. The framework is categorized into four levels: (1) molecular and cellular targets, (2) network‐ and systems‐level changes, (3) psychological processes, and (4) set and setting influences.

## 5 Considerations for Future Research

While there is rationale to support future psychedelic clinical trials targeting comorbid MDD and chronic pain, it is important to acknowledge several unique safety and feasibility considerations. There is substantial diversity in the etiology and clinical presentation of both MDD and chronic pain. MDD can be delineated into several subtypes based on onset, symptom profile, severity, and treatment response [134]. Chronic pain encompasses a wide range of emotional and physical experiences. Chronic pain can originate secondary to an illness or injury but it can also develop in the absence of an identifiable source. MDD and chronic pain disorders are also associated with high rates of additional comorbidities. Several physical illnesses including endocrine, musculoskeletal, and cardiovascular diseases are associated with MDD and chronic pain [135]. Common psychiatric comorbidities include substance use, somatoform, and anxiety disorders, as well as sleep disturbances and insomnia [136, 137]. Biopsychosocial factors further influence how symptoms are expressed, contributing to significant inter‐individual variability [138].

The diversity of MDD and chronic pain disorders has important implications for the design and conduct psychedelic clinical trials. Varying etiologies and symptom presentations can make it difficult to distinguish adverse drug reactions from symptoms of existing conditions. While clinical trials report psilocybin and other serotonergic psychedelics as well‐tolerated, medical or psychiatric comorbidities may influence the safety and efficacy of treatment through drug–drug, drug–disease, and disease–disease interactions [139]. Specifically, patients with MDD and chronic pain experience a high degree of polypharmacy, which often include serotonergic antidepressants (e.g., SSRIs) and narcotic analgesics (e.g., opioids) [5, 140, 141, 142]. In most psychedelic trials, including for MDD, participants are required to taper off concomitant serotonergic medications for the duration of the study and abstain from taking opioids for within 12 h prior to the intervention and 6 h after administration. These restrictions may introduce specific recruitment and safety issues, including an elevated risk of opioid withdrawal. To mitigate risks for this population, future psychedelic trials should consider implementing an enhanced informed consent process and a closely monitored safety plan, particularly during medication tapering and the acute phase of the intervention.

## 6 Discussion

Psychedelic therapies often produce profound and deeply meaningful experiences that emerge from a dynamic interplay between molecular, neural, psychological, and environmental domains. Serotonergic psychedelics activate the 5‐HT2A receptor, initiating several downstream cascades that impact neurogenesis, inflammatory cytokines, and large‐scale brain network connectivity. Psychological factors, particularly set and setting, play an important role in preparing individuals for the psychedelic experience and framing their therapeutic potential. Understanding the therapeutic action of psychedelics therefore requires an interdisciplinary approach to fully capture how these systems interact to produce antidepressant and analgesic effects.

While findings from this review highlight promising mechanisms, several limitations must be acknowledged. The selection of studies in this review was not systematic and may reflect selection bias. The evolving nature of the field also means that many included studies are small, open‐label, or exploratory, with many mechanistic insights being associative rather than causal. Considerable heterogeneity in methodologies, including varying doses, compounds, and outcome measures, further complicates direct comparison. Variability in therapeutic support, music, and the physical environment of the dosing sessions limits generalizability. It is also important to note that few trials directly assess patients with comorbid depression and pain, making extrapolation from single‐diagnosis populations necessary. Conclusions drawn from the existing literature should therefore be interpreted with caution.

Given the several shared mechanisms between depression and chronic pain, future research should prioritize clinical trials targeting this population. Incorporating biomarkers and neuroimaging techniques into larger and more diverse populations could clarify how molecular and neural processes interact to improve clinical outcomes. Future studies should also compare sub‐hallucinogenic and hallucinogenic dosing strategies to inform whether full psychedelic experiences are necessary for symptom relief. Further, more research is needed to understand the role of contextual factors. Experimental studies using dismantling designs or adjunctive psychotherapy arms may help clarify the relative contribution of psychosocial versus pharmacological effects. Finally, long‐term studies are needed to understand the durability of therapeutic effects and the ongoing role of integration support in sustaining psychological and functional improvements.

## 7 Conclusion

Psychedelics represent a promising therapeutic approach in the treatment of comorbid MDD and chronic pain. Although current findings are largely extrapolated from depression‐only or pain‐only populations, the wide range of proposed mechanisms provides a strong rationale for future clinical trials targeting those with an MDD and pain comorbidity. Clarifying the role of neurobiological mechanisms alongside contextual factors will be essential for translating the therapeutic potential of psychedelics into safe and effective interventions.

## Author Contributions

**Karim S. Ladha:** conceptualization, writing – review and editing, supervision. **Jordana Kazdan:** conceptualization, writing – original draft, writing – review and editing, data curation, visualization. **M. Ishrat Husain:** conceptualization, writing – review and editing, supervision.

## Funding

The authors have nothing to report.

## Ethics Statement

The authors have nothing to report.

## Conflicts of Interest

M. Ishrat Husain reports a relationship with Brain and Behavior Research Foundation that includes: funding grants; a relationship with Canadian Institutes of Health Research that includes: funding grants; a relationship with CAMH Foundation that includes: funding grants; a relationship with Grand Challenges Canada that includes: funding grants; a relationship with Physicians' Services Inc. Foundation that includes: funding grants; a relationship with University of Toronto that includes: funding grants; a relationship with Mindset Pharma that includes: consulting or advisory and equity or stocks; a relationship with PsychEd Therapeutics that includes: consulting or advisory; a relationship with Wake Network that includes: consulting or advisory; and a relationship with Compass Pathfinder Limited that includes: funding grants. Karim Ladha reports support from the Department of Anesthesiology and Pain Medicine at the University of Toronto (Merit Award), the Canadian Anesthesiologists' Society (Career Scientist Award), and the Evelyn Bateman Recipe Chair in Ambulatory Anesthesia and Women's Health at Women's College Hospital. Karim Ladha has previously received advisory board fees from Vectura Fertin Pharma and Merck.

## Data Availability

Data sharing is not applicable to this article as no datasets were generated or analyzed during this study.
